# A Domain Adaptation Sparse Representation Classifier for Cross-Domain Electroencephalogram-Based Emotion Classification

**DOI:** 10.3389/fpsyg.2021.721266

**Published:** 2021-07-29

**Authors:** Tongguang Ni, Yuyao Ni, Jing Xue, Suhong Wang

**Affiliations:** ^1^School of Computer Science and Artificial Intelligence, Changzhou University, Changzhou, China; ^2^School of Electrical Engineering, Xi'an Jiaotong University, Xi'an, China; ^3^Department of Nephrology, Affiliated Wuxi People's Hospital of Nanjing Medical University, Wuxi, China; ^4^Department of Clinical Psychology, The Third Affiliated Hospital of Soochow University, Changzhou, China

**Keywords:** electroencephalogram, domain adaptation, emotion classification, cross-subject, cross-dataset

## Abstract

The brain-computer interface (BCI) interprets the physiological information of the human brain in the process of consciousness activity. It builds a direct information transmission channel between the brain and the outside world. As the most common non-invasive BCI modality, electroencephalogram (EEG) plays an important role in the emotion recognition of BCI; however, due to the individual variability and non-stationary of EEG signals, the construction of EEG-based emotion classifiers for different subjects, different sessions, and different devices is an important research direction. Domain adaptation utilizes data or knowledge from more than one domain and focuses on transferring knowledge from the source domain (SD) to the target domain (TD), in which the EEG data may be collected from different subjects, sessions, or devices. In this study, a new domain adaptation sparse representation classifier (DASRC) is proposed to address the cross-domain EEG-based emotion classification. To reduce the differences in domain distribution, the local information preserved criterion is exploited to project the samples from SD and TD into a shared subspace. A common domain-invariant dictionary is learned in the projection subspace so that an inherent connection can be built between SD and TD. In addition, both principal component analysis (PCA) and Fisher criteria are exploited to promote the recognition ability of the learned dictionary. Besides, an optimization method is proposed to alternatively update the subspace and dictionary learning. The comparison of CSFDDL shows the feasibility and competitive performance for cross-subject and cross-dataset EEG-based emotion classification problems.

## Introduction

Emotion is the attitude experience and corresponding behavior response of human beings to objective things, which has an important influence on human behavior and mental health. How to accurately identify emotions has an important significance in practical application. For example, in the medical field, emotion recognition is helpful to guide and diagnose patients with mental diseases or expression disorders, and in the education field, different teaching methods according to the emotion of the listener can improve the teaching efficiency.

Emotion recognition using a variety of modal emotion signals has now gained a lot of attention from researchers. Typically, emotions can be perceived in the form of a variety of signals. One type of visual signal can be directly observed from external behavior and characteristics, such as facial expressions, voice intonation, body movements, etc. The other type is those physiological signals, such as electroencephalography (EEG), electromyography (EMG), electrocardiogram (ECG), skin conductance, pulse, heartbeat, skin temperature, and respiratory signals; however, facial expressions, voice, and other non-physiological signals are easily restricted by environmental or social factors. The emotional information transmitted by physiological signals is more objective and can reflect the psychological emotion more reliably (Zheng et al., 2019; Doma and Pirouz, 2020; Ni et al., 2020a).

**Figure 1:**
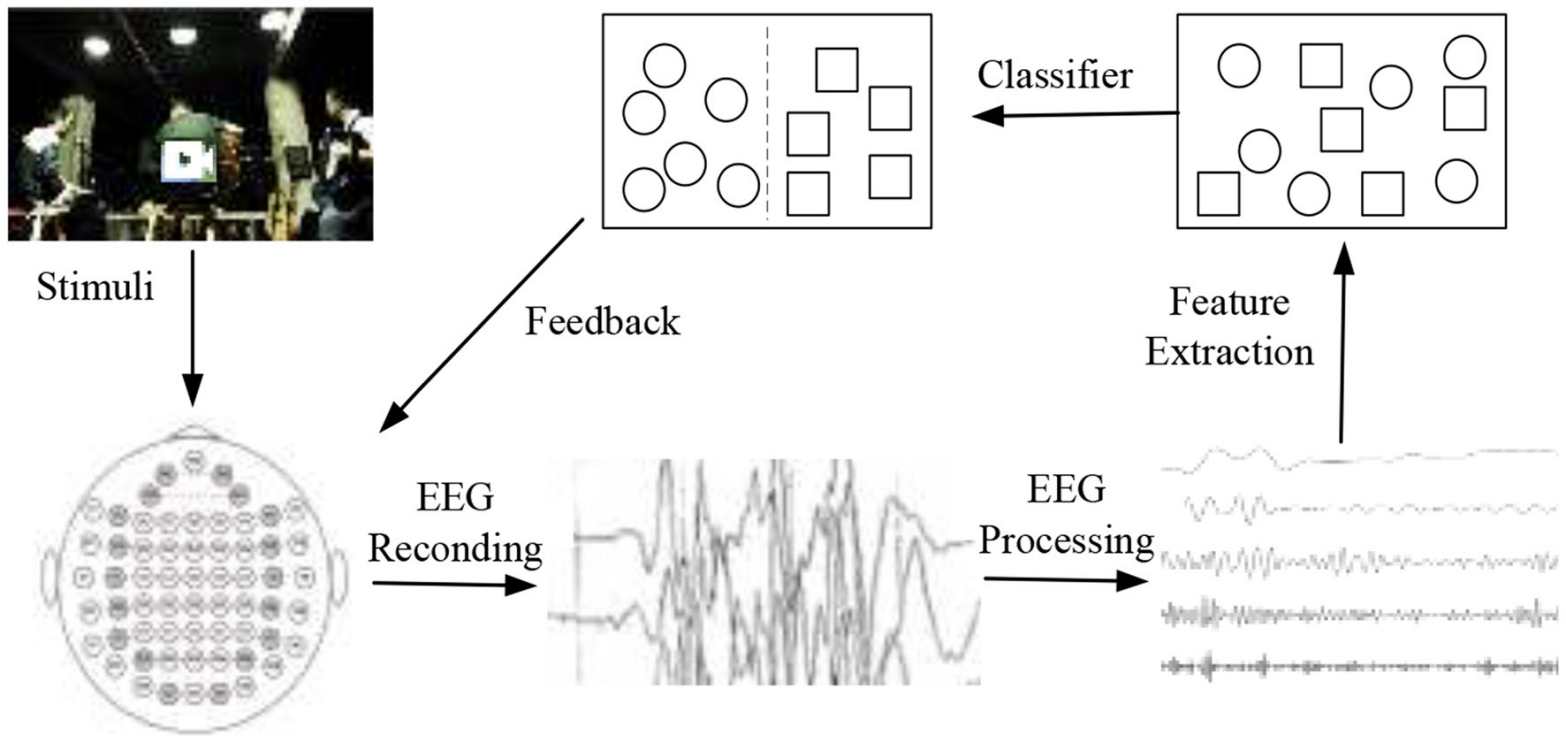
The illustration of EEG-based emotion classification in BCI.

Brain-computer interface (BCI) is a human-computer interaction system that provides a communication channel for human brain interaction with the external environment and without depending on the peripheral nervous system and muscles (Zhang and Wu, 2019; Liu et al., 2020; Ni et al., 2020b). EEG plays a dominant role in emotion recognition based on physiological signals. The illustration of EEG-based emotion classification in BCI is shown in Figure 1. The operation of emotion classification begins with the presentation of stimuli to the user, which induces specific emotions. The stimuli may be music, videos, and images, etc. During the session, EEG samples are recorded by EEG devices. The next step is usually to extract features from the recorded EEG and train a classifier. The final step is to test new EEG samples to classify emotion labels (Liu et al., 2018).

The existing EEG-based emotional classification in BCI requires a large amount of label data and a lot of time in the training phase. A relatively simple and direct method is to reuse previously collected EEG data and train a new classifier, without considering differences between individuals. These classification methods are based on the assumption that training and test data are independently and identically distributed. This assumption is often difficult to hold for BCI, because EEG signals have non-stationary characteristics, and the performance of the classifier fluctuates significantly between subjects and datasets. When the same classifier is applied to EEG data of other subjects or from other datasets, the performance will be significantly reduced.

Domain adaptation learning is a fast and effective solution for developing a classifier that selectively trains a new classifier in TD using auxiliary data (source domain, SD) and less training data in the new scenario (target domain, TD) (Fahimi et al., 2019; Ni et al., 2020c). Different from multi-task learning that aims to benefit the classifier in both source and target tasks, domain adaptation learning mainly aims to benefit the classifier in TD. For example, Yang et al. (2019) proposed a support vector machine (SVM) combined with the significance test and sequential backward selection strategy for cross-subject EEG-based emotional classification. Instead of utilizing features on raw EEG signals, this method analyzed and selected features based on the significant differences between positive and negative trials. Li et al. (2020a) proposed a two-stage multi-source semi-supervised transfer learning method, in which the work of the first stage was source domain selection and the second stage was to learn style transfer mapping. This method selected the appropriate sources and projected source data to the destination *via* an affine mapping, so that only a few labeled data was used in the calibration sessions. Subsequently, Li et al. (2020b) developed a joint distribution adaptation method for EEG-based emotion classification in cross-subject and cross-session scenarios. The label information in the SD was used to train the model, and it also took an important part in reducing the difference of conditional distribution. This method achieves domain adaptation by combining marginal distributions and conditional distributions in the framework of neural networks. Morioka et al. (2015) also developed a cross-subject and cross-session recognition method, which learned the common spatial bases underlying both SD and TD by using unsupervised dictionary learning. The spatial transforms technology was found to be efficient in extracted common brain activities. Lan et al. (2018) developed a domain adaptation method to reduce discrepancies across datasets and inter-subject variance. This method designed a linear transformation function to adapt subspaces feature to match the marginal distributions of SD and TD.

Although domain adaptation for EEG-based emotion classification has been extensively studied, most of the studies focus on cross-subject and cross-session adaptation within the same dataset, i.e., the samples of SD and TD came from the same dataset. Domain adaptation across datasets is more challenging. Because cross-dataset domain adaptation is restricted to the different datasets, EEG signals are collected from the different devices and different stimuli, etc. (Li et al., 2018; Yang et al., 2019; Cimtay and Ekmekcioglu, 2020).

It is our opinion that although the distribution of common characters in EEG signals shows differences between subjects and datasets, it is expected that there might be some certain common knowledge that is potentially independent of the subjects and datasets. In addition, the shared common knowledge could be preserved in a shared projection subspace. Thus, we propose a domain adaptation sparse representation classifier (DASRC) to address the EEG-based emotion classification in cross-subject and cross-dataset scenarios. We consider learning the common component in both SD and TD by exploring a common dictionary in a shared subspace. Thus, we adopt the local information preserved criterion to reduce the domain distribution discrepancies in the learned subspace. We learn the common domain-invariant dictionary, which builds a connection between SD and TD. In addition, the principal component analysis (PCA) and Fisher criteria are exploited in this model to promote the recognition ability of the learned classifier.

The main contribution of the study is as follows. First, DASRC exploits the common characteristics of EEG data in SD and TD to yield a domain-invariant dictionary in the shared subspace. It takes advantage of the local data information preserved in both SD and TD. This allows enhancing the domain adaptation in subspace. Second, using PCA and Fisher criteria, the objective function of DASRC is directly related to the classification rule. This strategy can promote the recognition ability in the domain-invariant subspace. Mathematically, an alternating optimization algorithm is proposed to solve the subspace and dictionary learning problem. Third, experiments on SJTU emotion EEG dataset (SEED) (Zheng and Lu, 2015) and dataset for emotion analysis using EEG, physiological and video signals (DEAP) (Koelstra et al., 2011) demonstrate that dictionary learning in subspace is effective and DASRC outperforms the advanced methods in cross-subject and cross-dataset scenarios.

## Background

Sparse representation is a data analysis method to estimate the sparse representation of measurable signals completely. It originated from neuroscience and has been used in signal processing, such as denoising and compression (Kanoga et al., 2019; Gu et al., 2020). In pattern recognition, sparse representation has also been proved to be suitable for classification. In sparse representation, the data matrix **Y** = [**y**_1_, **y**_2_, …, **y**_*n*_] can be decomposed into a linear combination of a few atoms on the dictionary,

(1) $$\begin{array}{l} \text{Y}\approx \text{D}\text{A}, \end{array}$$

where **D** is the dictionary matrix, and **A** is the coefficients matrix.

An adequate approximation makes **DA** the sparse representation as a reasonable estimation of**Y**. Based on this concept, Equation (1) can be rewritten as follows:

(2) $$\begin{array}{l} \arg\underset{\text{D},\text{A}}{\min}\;||\text{Y}-\text{D}\text{A}|{|}_{F}^{2},\\ s.t.\;\forall i,{\left||{\text{a}}_{i}|\right|}_{0}\leq l_{0}, \end{array}$$

where *l*_0_ represents the sparsity constraint, **a**_*i*_ is the sparse coefficient vector to represent **y**_*i*_ over D. *K*-singular value decomposition (KSVD) algorithm is one of the most representative to solve Equation (2), in which the sparse coding and dictionary are updated alternately (Aharon et al., 2006).

## Domain Adaptation Sparse Representation Classifier

**Figure 2:**
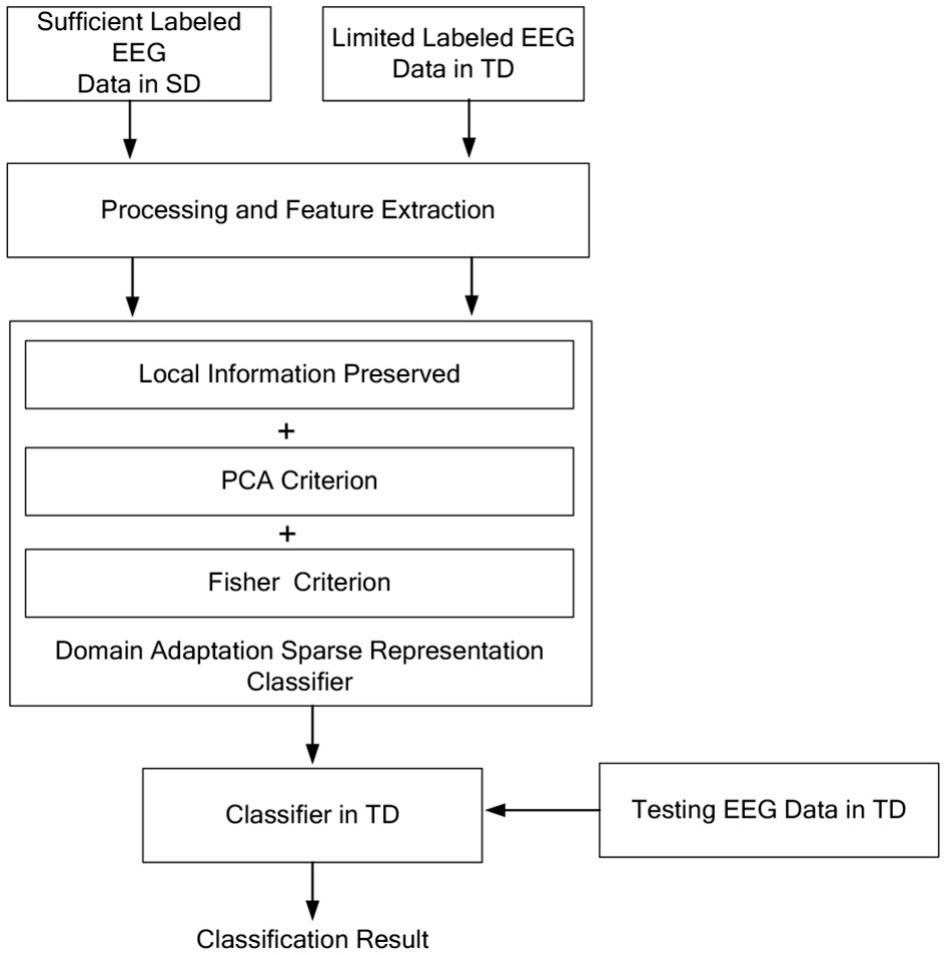
The main idea of the proposed model.

In this study, we consider the EEG data from two different domains SD and TD. The SD is with sufficient labeled samples ${\text{Y}}^{s}=\left[{\text{y}}_{1}^{s},{\text{y}}_{2}^{s},...,{\text{y}}_{n_{s}}^{s}\right]\in R^{d\times n_{s}}$ and the TD is with limited labeled samples ${\text{Y}}^{t}=\left[{\text{y}}_{1}^{t},{\text{y}}_{2}^{t},...,{\text{y}}_{n_{t}}^{t}\right]\in R^{d\times n_{t}}$, such that the data distribution is *P*(**Y**^*s*^ ≠ **Y**^*t*^) and $P\left({\text{Y}}^{s}|{\tilde{\text{L}}}^{s}\right)\approx P\left({\text{Y}}^{t}|{\tilde{\text{L}}}^{t}\right)$, where ${\tilde{\text{L}}}^{s}$ and ${\tilde{\text{L}}}^{t}$ are the class label set of samples in SD and TD, respectively. The main idea of the proposed model is shown in Figure 2.

### Local Information Preserved in DASRC

Because the EEG signals are collected from different domains, domain discrepancies exist between SD and TD. Usually, directly adapting the existing classifier in SD may perform poorly to new samples in TD. Domain adaptation is adopted in this study to find a latent and domain-invariant subspace and maps **Y**^*s*^ and **Y**^*t*^ by projection matrixes **M**^*s*^ and **M**^*t*^, respectively. In this domain-invariant subspace, the discrepancy between SD and TD is reduced. Finally, we can train a new classifier for TD in the subspace with the help of discriminative knowledge from labeled samples in SD.

Domain adaptation method should strive to preserve the class distribution and local characteristics of training samples. Therefore, the local geometric structure of the samples is considered, so that the samples in SD and TD can be validly represented in the domain-invariant subspace. We construct the similarity matrixes **G**^*s*^ and **G**^*t*^ of SD and TD by *k*-nearest neighbor graphs, respectively. The elements in **G**^*s*^ and **G**^*t*^ can be computed by the following equations,

(3) $$G_{i,j}^{s}=\{\begin{array}{ll} \exp(-\frac{{\left\|y_{i}^{s}-y_{j}^{s}\right\|}_{2}}{\sigma }) & if\;y_{i}^{s}\in KNN(y_{j}^{s})\;\\ \;0 & else \end{array}$$

(4) $$G_{i,j}^{t}=\{\begin{array}{cc} 1 & if\;y_{i}^{t}\;and\;y_{j}^{t}\;are\;ofthe\;same\;class\\ \;0 & else \end{array}$$

where $KNN\left({\text{y}}_{j}^{s}\right)$ is a set that contains *k* nearest neighbor samples of ${\text{y}}_{j}^{s}$. The element $G_{i,j}^{s}$ presents the similarity between the *i*th and *j*th samples in SD. As one of the most commonly used similarity metrics in graph learning, the Gaussian kernel is adopted in Equation (3). σ is the kernel parameter. Since the number of samples in TD is insufficient, Equation (4) ensures that the limited number of samples in TD is assigned with a given one-to-one weight. Therefore, we construct the local preserved constraint to maintain the intra-domain local information.

(5) $$\begin{array}{l} \underset{{\text{M}}^{s},{\text{M}}^{t}}{\min}J_{1}=\underset{i,j}{\sum }G_{i,j}^{s}||\left({\text{M}}^{s}{\text{Y}}_{i}^{s}-{\text{M}}^{s}{\text{Y}}_{j}^{s}\right)|{|}_{2}^{2}\\ \;+\underset{i,j}{\sum }G_{i,j}^{t}||\left({\text{M}}^{t}{\text{Y}}_{i}^{t}-{\text{M}}^{t}{\text{Y}}_{j}^{t}\right)|{|}_{2}^{2}. \end{array}$$

### PCA Criterion in DASRC

In addition, with the aim of classification, the discriminative knowledge of SD and TD should be enforced in subspace projection. We consider **M**^*s*^ and **M**^*t*^ are the bases of the subspaces based on the PCA criterion for SD and TD. Following Gong et al. (2019) and Ma et al. (2011), the PCA criterion is used to preserve discriminative knowledge in the subspace. To this end, we minimize the following optimization problem:

(6) $$\begin{array}{l} \;\underset{M^{s},M^{t}}{\min}J_{2}=\;-{\text{M}}^{s}{\text{Y}}^{s}{\left({\text{Y}}^{s}\right)}^{T}{\left({\text{M}}^{s}\right)}^{T}-{\text{M}}^{t}{\text{Y}}^{t}{\left({\text{Y}}^{t}\right)}^{T}{\left({\text{M}}^{t}\right)}^{T},\\ s.t.\;{\text{M}}^{s}{\left({\text{M}}^{s}\right)}^{T}=\text{I},\\ \;{\text{M}}^{t}{\left({\text{M}}^{t}\right)}^{T}=\text{I}. \end{array}$$

where I is the identity matrix.

The Laplacian matrixes are denoted as **L**^*s*^ = **G**^*s*^ − **W**^*s*^ and **L**^*t*^ = **G**^*t*^ − **W**^*t*^, where **G**^*s*^ and **G**^*t*^ are diagonal matrixes whose elements in the principal diagonal are defined as ${\text{W}}_{i,i}^{s}=\underset{j}{\sum }{\text{G}}_{i,j}^{s}$and ${\text{W}}_{i,i}^{t}=\underset{j}{\sum }{\text{G}}_{i,j}^{t}$, the term *J*_1_ + *J*_2_ can be written as

(7) $$\begin{array}{l} \underset{M^{s},M^{t}}{\min}J_{1}+J_{2}=Tr(M^{s}Y^{s}(L^{s}-\alpha I){\left(Y^{s}\right)}^{T}{\left(M^{s}\right)}^{T}\\ +Tr(M^{t}Y^{t}(L^{t}-\alpha I){\left(Y^{t}\right)}^{T}{\left(M^{t}\right)}^{T},\\ s.t.\;M^{s}{\left(M^{s}\right)}^{T}=I,\\ \;M^{t}{\left(M^{t}\right)}^{T}=I. \end{array}$$

where α is the regularization parameter.

### Fisher Criterion in DASRC

To train the discriminative dictionary D in the projection space, where the learned dictionary can sparsely represent samples from each class in SD and TD, the representation errors of intraclass and interclass in SD and TD are required to be minimized and maximized, respectively. Inspired by the Fisher criterion (Peng et al., 2020), the ratio of intra-class scatter to inter-class scatter are minimized on the coding coefficients in SD and TD as follows:

(8) $$\begin{array}{l} \underset{{\text{M}}^{s},{\text{M}}^{t},\text{D}}{\arg\min}J_{3}=\frac{\underset{j}{\sum }||{\text{M}}^{s}{\text{y}}_{j}^{s}-\text{D}\varphi_{l_{j}^{s}}\left(\alpha_{j}^{s}\right)|{|}_{F}^{2}+\underset{j}{\sum }||{\text{M}}^{t}{\text{y}}_{j}^{t}-\text{D}\varphi_{l_{j}^{t}}\left(\alpha_{j}^{t}\right)|{|}_{F}^{2}\;}{\underset{j}{\sum }||{\text{M}}^{s}{\text{y}}_{j}^{s}-\text{D}\theta_{l_{j}^{s}}\left(\alpha_{j}^{s}\right)|{|}_{F}^{2}+\underset{j}{\sum }||{\text{M}}^{t}{\text{y}}_{j}^{t}-\text{D}\theta_{l_{j}^{t}}\left(\alpha_{j}^{t}\right)|{|}_{F}^{2}},\\ s.t.\;||d_{k}|{|}_{2}^{2}\leq 1,\;\forall k \end{array}$$

where the function φ_*l*_*j*__() returns the coefficient vectors of the same class of *y_j_*, and the function θ_*l*_*j*__() returns the coefficient vectors of the different class of *y*_*j*_.

To simplify Equation (8), we denote

(9.1) $$\Theta^{s}=[\varphi_{l_{1}^{s}}(\alpha_{1}^{s}),\varphi_{l_{2}^{s}}(\alpha_{2}^{s}),\ldots ,\varphi_{l_{n_{s}}^{s}}(\alpha_{n_{s}}^{s})],$$

(9.2) $$\Theta^{t}=[\varphi_{l_{1}^{t}}(\alpha_{1}^{t}),\varphi_{l_{2}^{t}}(\alpha_{2}^{t}),\ldots ,\varphi_{l_{n_{t}}^{t}}(\alpha_{n_{t}}^{t})],$$

(9.3) $$\Delta^{s}=[\theta_{l_{1}^{s}}(\alpha_{1}^{s}),\theta_{l_{2}^{s}}(\alpha_{2}^{s}),\ldots ,\theta_{l_{n_{s}}^{s}}(\alpha_{n_{s}}^{s})],$$

(9.4) $$\Delta^{t}=[\theta_{l_{1}^{t}}(\alpha_{1}^{t}),\theta_{l_{2}^{t}}(\alpha_{2}^{t}),\ldots ,\theta_{l_{n_{t}}^{t}}(\alpha_{n_{t}}^{t})],$$

Equation (8) can be re-written as

(10) $$\begin{array}{l} \underset{M^{s},M^{t},D}{\arg\min}J_{3}=\frac{{\left\|\left(M^{s}Y^{s}-D\Theta^{s}\right)\right\|}_{F}^{2}\text{+}{\left\|\left(M^{t}Y^{t}-D\Theta^{t}\right)\right\|}_{F}^{2}}{{\left\|\left(M^{s}Y^{s}-D\Delta^{s}\right)\right\|}_{F}^{2}\text{+}{\left\|\left(M^{t}Y^{t}-D\Delta^{t}\right)\right\|}_{F}^{2}},\\ s.t.\;{\left\|d_{k}\right\|}_{2}^{2}\leq 1,\;\forall k \end{array}$$

when the minimization problem of *J*_3_ is solved, a shared dictionary is learned to establish an intrinsic relationship between different domains so that the discrimination information learned from SD can be transferred to TD in a cross-domain scenario.

### The DASRC Model

For cross-domain EEG-based emotion classification, we take into account three objective functions *J*_1_, *J*_2_, and *J*_3_ together, joint constraints on the local information preserved, PCA and Fisher criteria to optimize the shared dictionary and domain-specific projections. Thus, the optimization problem of DASRC can be formulated as

(11) $$\begin{array}{l} \underset{{\text{M}}^{s},{\text{M}}^{t},\text{D},{\text{A}}^{s},{\text{A}}^{t}}{\arg\min}\;J_{1}+J_{2}+J_{3},\\ s.t.\;{\text{M}}^{s}{\left({\text{M}}^{s}\right)}^{T}=\text{I},\\ \;{\text{M}}^{t}{\left({\text{M}}^{t}\right)}^{T}=\text{I}. \end{array}$$

To see all the components clearly, Equation (11) is expanded by

(12) $$\begin{array}{l} \underset{M^{s},M^{t},D}{\arg\min}\frac{{\left\|\left(M^{s}Y^{s}-D\Theta^{s}\right)\right\|}_{F}^{2}\text{+}{\left\|\left(M^{t}Y^{t}-D\Theta^{t}\right)\right\|}_{F}^{2}}{{\left\|\left(M^{s}Y^{s}-D\Delta^{s}\right)\right\|}_{F}^{2}\text{+}{\left\|\left(M^{t}Y^{t}-D\Delta^{t}\right)\right\|}_{F}^{2}}\\ +Tr(M^{s}Y^{s}(L^{s}-\alpha I){\left(Y^{s}\right)}^{T}{\left(M^{s}\right)}^{T}\\ +Tr(M^{t}Y^{t}(L^{t}-\alpha I){\left(Y^{t}\right)}^{T}{\left(M^{t}\right)}^{T},\\ s.t.\;M^{s}{\left(M^{s}\right)}^{T}=I,\\ \;M^{t}{\left(M^{t}\right)}^{T}=I. \end{array}$$

Let $\tilde{\text{M}}=\left[{\text{M}}^{s},{\text{M}}^{t}\right]$, $\tilde{\text{Y}}=\left[\begin{array}{cc} {\text{Y}}^{s} & 0\\ 0 & {\text{Y}}^{t} \end{array}\right]$, $\tilde{\Theta }=\left[\Theta^{s},\Theta^{t}\right]$, $\tilde{\Delta }=\left[\Delta^{s},\Delta^{t}\right]$, $\tilde{\text{L}}=\left[\begin{array}{cc} {\text{L}}^{s} & 0\\ 0 & {\text{L}}^{t} \end{array}\right]$, the simplified formulation of Equation (12) can be approximately written as

(13) $$\begin{array}{l} \underset{\tilde{M},D}{\min}\frac{{\left\|\tilde{M}\tilde{Y}-D\tilde{\Theta }\right\|}_{F}^{2}}{{\left\|\tilde{M}\tilde{Y}-D\tilde{\Delta }\right\|}_{F}^{2}}+\;Tr(\tilde{M}\tilde{M}(L-\alpha I){\tilde{Y}}^{T}{\tilde{M}}^{T},\\ s.t.\;\tilde{M}{\tilde{M}}^{T}=I. \end{array}$$

### Optimization of the Objective Function

Equation (13) becomes a least square problem with quadratic constraints and could be solved by many methods. The Lagrangian of the optimization problem in Equation (13) is

(14) $$\begin{array}{l} F(\tilde{M},D\text{,}\beta ,\gamma )={\left\|\tilde{M}\tilde{Y}-D\tilde{\Theta }\right\|}_{F}^{2}\text{+ }Tr(\tilde{M}\tilde{Y}(L-\alpha I){\tilde{Y}}^{T}{\tilde{M}}^{T}\\ \;-\beta {\left\|\tilde{M}\tilde{Y}-D\tilde{\Delta }\right\|}_{F}^{2}-\gamma (\tilde{M}{\tilde{M}}^{T}-I), \end{array}$$

where β and γ are Lagrange multipliers.

We use the alternating optimization method to solve Equation (14). When fixing **D**, β, and γ, we take the first-order partial derivatives of Equation (14) over $\tilde{\text{M}}$,

(15) $$\begin{array}{l} F^{\prime }(\tilde{M})=\tilde{M}\tilde{Y}{\tilde{Y}}^{T}-D\tilde{\Theta }{\tilde{Y}}^{T}+\tilde{M}\tilde{Y}(L-\alpha I){\tilde{Y}}^{T}\\ \;-\beta (\tilde{M}\tilde{Y}{\tilde{Y}}^{T}-D\tilde{\Delta }{\tilde{Y}}^{T}\text{)}-\gamma \tilde{M}, \end{array}$$

and the optimal $\tilde{\text{M}}$ can be computed in the closed-form

(16) $$\begin{array}{l} \tilde{M}=(D\tilde{\Theta }{\tilde{Y}}^{T}-\beta D\tilde{\Delta }{\tilde{Y}}^{T}\text{)(}\tilde{Y}{\tilde{Y}}^{T}+\tilde{Y}(L-\alpha I){\tilde{Y}}^{T}\\ \;-\beta \tilde{Y}{\tilde{Y}}^{T}-\gamma I{\text{)}}^{-1}. \end{array}$$

When fixing $\tilde{\text{M}}$, β, and γ, we take the first-order partial derivative of Equation (14) over **D**,

(17) $$\begin{array}{l} F^{\prime }\left(\text{D}\right)=-\tilde{\text{M}}\tilde{\text{Y}}{\tilde{\Theta }}^{T}\text{+}\text{D}\tilde{\Theta }{\tilde{\Theta }}^{T}\text{+}\beta \tilde{\text{M}}\tilde{\text{Y}}{\tilde{\Delta }}^{T}-\beta \text{D}\tilde{\Delta }{\tilde{\Delta }}^{T}, \end{array}$$

and the optimal **D** can be computed in the closed-form

(18) $$\begin{array}{l} \text{D}\text{=(}\beta \tilde{\text{M}}\tilde{\text{Y}}{\tilde{\Delta }}^{T}-\tilde{\text{M}}\tilde{\text{Y}}{\tilde{\Theta }}^{T}\text{)(}\tilde{\Theta }{\tilde{\Theta }}^{T}-\beta \tilde{\Delta }{\tilde{\Delta }}^{T}{\text{)}}^{-1}. \end{array}$$

When fixing $\tilde{\text{M}}$, **D**, and γ, we take the first-order partial derivative of Equation (14) over β,

(19) $$\begin{array}{l} F^{\prime }\left(\beta \right)=-||\tilde{\text{M}}\tilde{\text{Y}}-\text{D}\tilde{\Delta }|{|}_{F}^{2}, \end{array}$$

Then β can be optimized by

(20) $$\begin{array}{l} \beta_{new}=\beta +\lambda_{\beta }\frac{F\left(\beta \right)}{F^{\prime }\left(\beta \right)}, \end{array}$$

where λ_β_ is the length size.

When fixing $\tilde{\text{M}}$, **D**, and β, we take the first-order partial derivative of Equation (14) over γ,

(21) $$\begin{array}{l} F^{\prime }\left(\gamma \right)=\text{I}-\tilde{\text{M}}{\tilde{\text{M}}}^{T}, \end{array}$$

Then γ can be optimized by,

(22) $$\begin{array}{l} \gamma_{new}=\gamma +\lambda_{\gamma }\frac{F\left(\gamma \right)}{F^{\prime }\left(\gamma \right)}, \end{array}$$

where λ_γ_ is the length size.

The proposed DASRC model is given in Algorithm 1.

**Algorithm 1:** DASRC: domain adaptation sparse representation classifier.

Input: Source domain dataset **Y**^*s*^ and target domain **Y**^*t*^;
Output: The dictionary **D** and subspace $\tilde{\text{M}}$;
1: Initialize **M**^*s*^ and **M**^*t*^ using PCA method and D using KSVD algorithm;
2: Construct *k*-nearest neighbor graphs in SD and TD;
3: Construct matrixes $\tilde{\text{Y}}$ and $\tilde{\text{L}}$;
While
4: Construct matrixes $\tilde{M}$,$\tilde{\Theta }$, and $\tilde{\Delta }$;
5. Update $\tilde{\text{M}}$ using Equation (16);
6: Update **D** using Equation (18);
7: Update β using Equations (19)–(20);
8: Update γ using Equations (21)–(22);
Return $\tilde{\text{M}}$ and D.

### Testing

With all the optimization steps discussed above, we summarize the optimization procedure of the DASRC model in Algorithm 1. When the projection matrix $\tilde{\text{M}}$ and the shared dictionary D are obtained by Algorithm 1, we use the following step to recognize the new EEG signal y in TD. The sparse coefficient vector **α** over dictionary D can be solved as,

(23) $$\underset{\alpha }{\min}\;{\left\|\tilde{M}y-D\alpha \right\|}_{2}^{2}+\mu {\left\|\alpha \right\|}_{2}^{2},$$

The sparse coefficient vector **α** can be obtained as

(24) $$\begin{array}{l} \alpha ={\left({\text{D}}^{T}\text{D}+\mu \text{I}\right)}^{-1}{\text{D}}^{T}\tilde{\text{M}}y, \end{array}$$

The classification label of x can be derived as

(25) $$\text{label(}y)=\arg\;\underset{i}{\min}\;\{{\left\|\tilde{M}y-D\varphi_{i}(\alpha )\right\|}_{2}^{2}/{\left\|\varphi_{i}(\alpha )\right\|}_{2}^{2}\},i=1,2,\ldots ,C$$

where *C* is the number of classes.

## Experiment

### Datasets and Experimental Settings

We conduct the experiments to evaluate the efficacy of the proposed classifier on two public EEG emotion datasets, SEED and DEAP. The EEG signals in the SEED dataset are recorded from 15 participants across three different sessions. Their emotions are stimulated by the Chinese file clips using an ESI NeuroScan system with 62-channel electrodes at a sampling rate of 1,000 Hz. Each film clip is related to three emotions as positive, neutral, or negative, and each emotion has five corresponding film clips. The EEG signals in the DEAP dataset are recorded from 32 participants by watching 40 videos with 32-channel electrodes. DEAP dataset labels the valence and arousal rating scores from 1 to 9, which is closely related to emotions. We manually label the valence values above 4.5 as positive and the values smaller than 4.5 as negative. For a comprehensive study, we extract four different features in terms of time analysis, frequency analysis, and non-linear analysis for each EEG channel. Time analysis includes mean absolute value (MAV) (Shim et al., 2016), frequency analysis includes power spectral density (PSD) (Jenke et al., 2014), and non-linear analysis includes fractal dimension (FD) (Li et al., 2019) and differential entropy (DE) (Zheng and Lu, 2015). The size of the extracted feature is 10 dimensions for each channel.

We evaluate the DASRC model on cross-subject and cross-dataset scenarios. We compared DASRC with two baseline methods and four domain adaptation methods. For the baseline methods, we compared the DASRC with label consistent K-SVD (LC-KSVD) (Jiang et al., 2013) and SVM (Cortes and Vapnik, 1995). For these two methods, the training data in SD and TD are combined as the input samples. The Gaussian kernel is used in SVM, and the kernel and penalty parameters are searched in the grid {10^−3^, 10^−2^,…, 10^3^}. The number of atoms in each class is selected in {50, 60,…, 200}. We compared the DASRC model with four domain adaptation methods, including transfer component analysis (TCA) (Pan et al., 2011), adaptive subspace feature matching (ASFM) (Chai et al., 2017), maximum mean discrepancy (MMD) (Sejdinovic et al., 2013), maximum independence domain adaptation (MIDA) (Yan et al., 2018). The latent dimension in MIDA and TCA is determined by searching the grid {20, 30,…, 100}. The subspace dimension in ASFM is set as 70. The threshold parameter in ASFM was set at 0.45. In DASRC, the subspace dimension is determined by searching the grid {20, 30,…, 100}. The number of atoms in each class is selected in {10, 15, 20, 25, 30, 35}. All the algorithms are implemented in MATLAB.

### Cross-Subject EEG-Based Emotion Classification

For cross-subject evaluation, one subject is left out as the test subject, and the remaining different subjects are used as training data to feed the model. In the SEED dataset, one subject contains 925 samples in each session. We randomly select 300 samples from each subject and combine them as training data. We repeat the procedure 10 times.

First, we evaluate how the classification accuracy of the classifier varies with the different features. For each subject, Table 1 depicts the experimental results of mean accuracies and SD on the SEED dataset. We can see that for DASRC, the classification accuracy is relatively stable, and the value of SD is acceptable. Meanwhile, the classification accuracy of some subjects is relatively high and of some subjects is relatively low. DE and FD features achieve better performance than PSD and MAV features.

**Table 1:** Cross-subject accuracy % (std %) of DASRC using different features on SEED dataset.

	**PSD**	**FD**	**DE**	**MAV**
Subject 1	73.76	79.43	79.84	74.02
	(4.56)	(6.33)	(5.24)	(5.61)
Subject 2	74.54	80.01	80.36	74.89
	(5.63)	(4.24)	(5.23)	(5.74)
Subject 3	63.99	69.35	69.49	64.37
	(5.34)	(6.17)	(6.02)	(7.26)
Subject 4	83.71	89.31	89.97	84.28
	(6.49)	(7.11)	(6.13)	(5.18)
Subject 5	73.29	78.23	78.62	73.95
	(6.69)	(7.11)	(7.13)	(7.07)
Subject 6	86.47	90.32	90.83	86.99
	(7.15)	(6.98)	(7.31)	(6.86)
Subject 7	67.85	72.06	72.65	67.82
	(6.49)	(6.75)	(6.71)	(6.66)
Subject 8	75.02	70.26	70.98	75.42
	(7.04)	(6.48)	(7.01)	(6.87)
Subject 9	79.66	84.09	84.26	79.95
	(7.42)	(7.16)	(7.05)	(7.04)
Subject 10	70.91	76.35	76.94	71.16
	(6.89)	(7.12)	(7.30)	(6.99)
Subject 11	74.90	80.14	80.47	75.25
	(7.73)	(7.56)	(7.49)	(7.58)
Subject 12	75.73	80.25	80.41	75.97
	(7.29)	(7.33)	(7.48)	(7.80)
Subject 13	81.28	86.45	86.78	81.44
	(7.31)	(7.45)	(7.68)	(7.54)
Subject 14	75.12	80.11	80.51	75.43
	(6.43)	(6.37)	(6.39)	(6.97)
Subject 15	75.32	81.26	81.93	75.75
	(6.32)	(6.27)	(6.66)	(6.74)
Mean	75.44	79.84	80.27	75.78
	(6.57)	(6.73)	(6.73)	(6.80)

Then, DASRC is compared with two baseline methods and four domain adaptation methods. Table 2 depicts the experimental results of mean accuracies of all models using PSD, MAV, DE, and FD features. From Table 2, we can see that the classification accuracies of all methods on DE and FD features are higher than PSD and MAV features. It may suggest that non-linear analysis features may be more suitable when compared to EEG-based emotion classification. The performance of the DE feature is better than that of the FD feature, and the best results in all methods are obtained using the DE feature.

**Table 2:** Cross-dataset accuracy % (std %) of DASRC using different features on SEED→ DEAP.

	**PSD**	**FD**	**DE**	**MAV**
SVM	33.34	35.75	36.66	33.94
	(8.04)	(9.00)	(8.36)	(9.10)
LC-KSVD	33.77	35.61	37.97	33.80
	(7.34)	(8.08)	(8.85)	(8.78)
TCA	41.69	44.12	45.08	41.90
	(7.27)	(9.35)	(9.01)	(9.70)
MIDA	43.61	45.13	45.86	42.96
	(9.50)	(10.81)	(8.52)	(6.65)
ASFM	44.67	47.14	47.97	44.88
	(8.96)	(9.04)	(10.48)	(8.66)
MMD	46.71	50.66	50.54	47.22
	(9.89)	(10.36)	(8.99)	(9.80)
DASRC	**50.57**	**53.26**	**53.54**	**50.60**
	(9.30)	(8.86)	(8.77)	(9.89)

*The bold values in Tables 2, 3 mean the best values in comparison experiments*.

**Figure 3:**
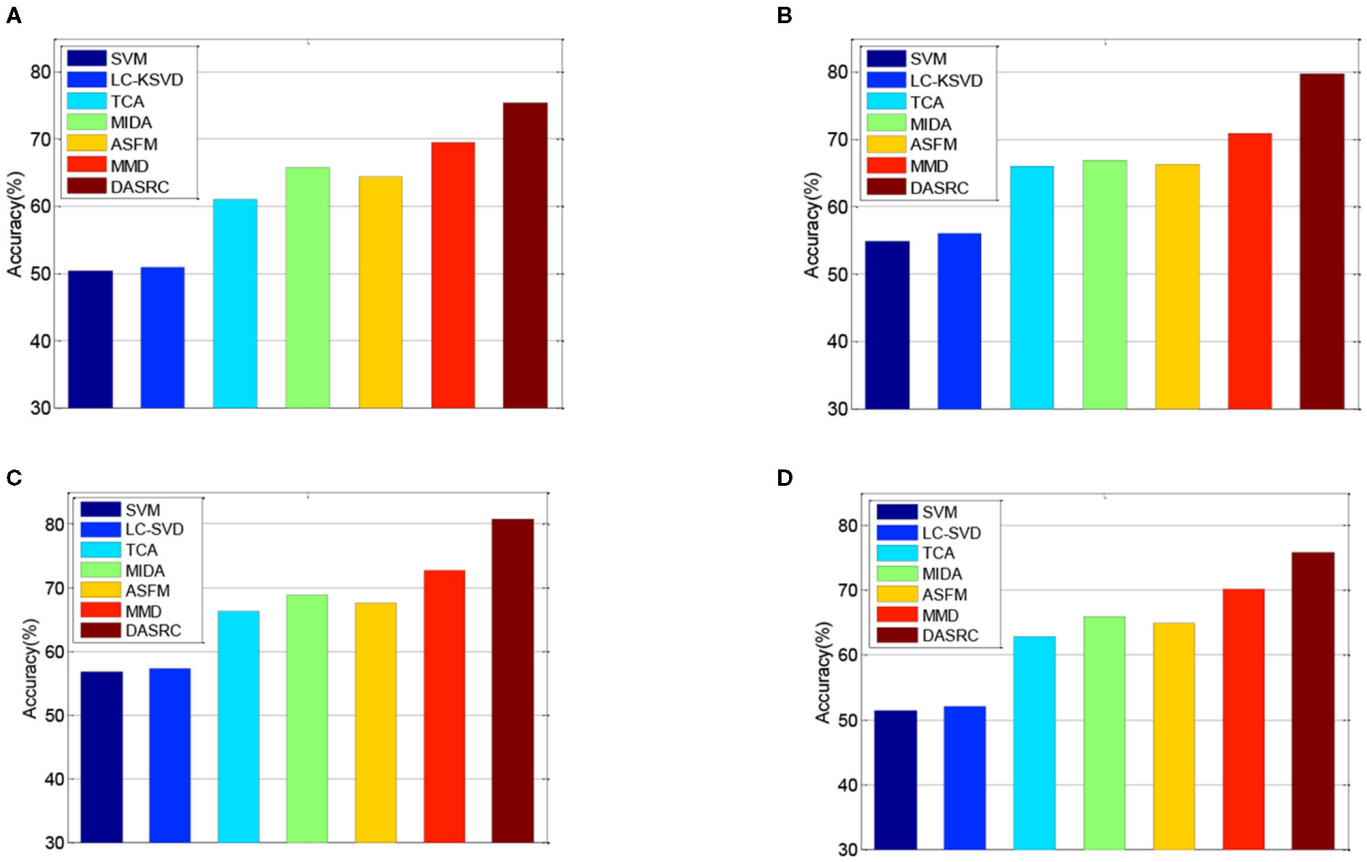
Cross-subject accuracy on SEED dataset with different features, **(A)** PSD, **(B)** FD, **(C)** DE, **(D)** MAV.

**Figure 4:**
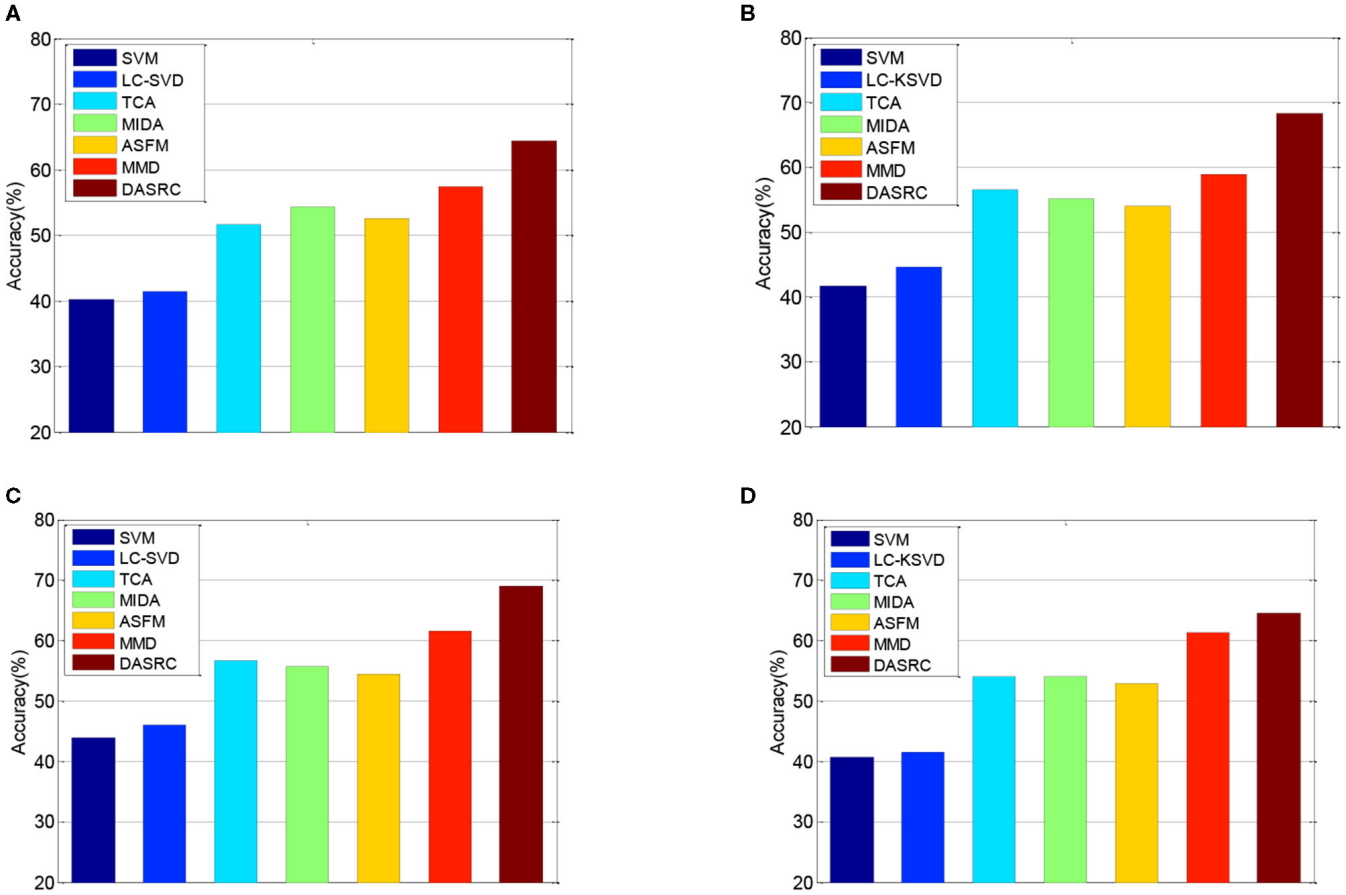
Cross-subject accuracy on DEAP dataset with different features, **(A)** PSD, **(B)** FD, **(C)** DE, **(D)** MAV.

In the DEAP dataset, one subject contains 180 samples. As such, we randomly select 100 samples from each subject and the training set contains 3,100 samples. Figure 3 shows the classification accuracies of six comparison methods on the SEED dataset when four different kinds of features (PSD, MAV, DE, and FD) are used. Figure 4 shows the accuracy results of five comparison methods on the DEAP dataset for positive and negative classes. According to the experimental results, we can see that first, single-domain classification methods SVM and LC-KSVD cannot obtain satisfactory classification performance in subject-to-subject scenarios on SEED and DEAP datasets. After all, they are not proposed to address the cross-domain data scenarios. Second, among the domain adaptation methods, the proposed DASRC model based on shared dictionary and subspace learning perform better than the methods using some other shared components. The main factor is that the shared dictionary can learn more discriminative knowledge to encode the EEG signals. Third, the accuracies of all models on the DEAP dataset are lower than those obtained on the SEED dataset. It may be the reason that the labeling quality of EEG signals in DEAP is poorer.

### Cross-Dataset EEG-Based Emotion Classification

For cross-dataset evaluation, the SD and TD are from different datasets. We perform comparison experiments across SEED and DEAP datasets. According to the given exact threshold, the samples in the DEAP dataset are divided into positive and negative classes, which correspond to the positive and negative classes of the SEED dataset. As the domain adaptation method requires the same feature space in SD and TD, we use the 32 channels shared between DEAP and SEED. In two experiments, we randomly select 3,000 samples in SD for training and 2,000 samples in TD for testing. We, then, repeat the procedure 10 times. Tables 2, 3, respectively, show the accuracy results of five comparison methods on SEED→ DEAP and DEAP→ SEED, when PSD, MAV, DE, and FD features are used. According to the experimental results, we can see that first, the performance of single-domain and domain adaptation methods exhibit evident differences, with three domain adaptation methods show significant improvements in classification accuracy. Second, DASRC is the best-performing classifier in two cross-dataset EEG emotion classifications. DASRC achieves 16.88% accuracy gains over the compared single-domain methods and 8.70% accuracy gains over the compared domain adaptation methods. The reason is that single-domain methods fail to reduce the domain shift. On the contrary, DASRC learns the shared dictionary to build the connection between SD and TD such that the discriminative knowledge from SD can be transferred to TD. In addition, besides the advantage of the cross-domain Fisher criterion and local information preserved technology, the recognition ability also promotes the classification performance of DASRC.

**Table 3:** Cross-dataset accuracy % (std %) of DASRC using different features on DEAP→ SEED.

	**PSD**	**FD**	**DE**	**MAV**
SVM	48.87	51.05	51.66	48.94
	(9.00)	(9.43)	(8.57)	(8.08)
LC-KSVD	49.30	51.73	51.97	49.86
	(9.76)	(10.72)	(7.70)	(6.68)
TCA	56.08	58.57	58.32	56.55
	(8.02)	(8.83)	(9.11)	(7.06)
MIDA	57.96	59.21	59.99	58.31
	(8.41)	(8.64)	(7.15)	(9.61)
ASFM	58.77	61.74	62.01	59.76
	(9.68)	(7.45)	(7.40)	(8.05)
MMD	61.04	63.60	63.89	61.25
	(8.82)	(7.00)	(8.64)	(7.24)
DASRC	**62.75**	**64.74**	**64.97**	**62.71**
	(8.20)	(7.33)	(7.67)	(7.05)

### Parameter Analysis

**Figure 5:**
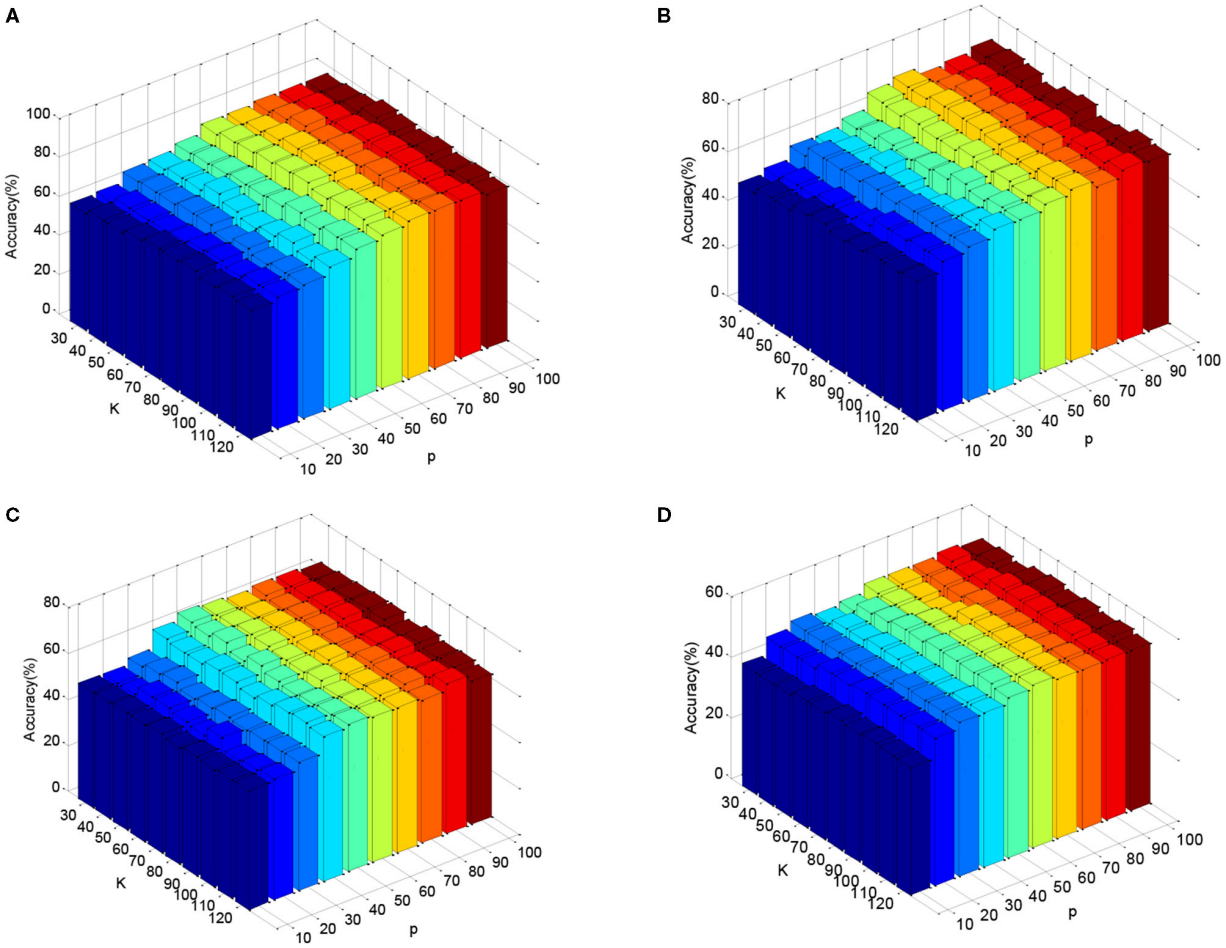
Cross-subject accuracy on DEAP dataset with different parameters p and K using features: **(A)** PSD, **(B)** FD, **(C)** DE, **(D)** MAV.

In this subsection, we validate the DASRC in cross-subject and cross-dataset scenarios. The subspace dimensions, *p*, and size of dictionary atoms, *K*, are the key parameters, and they are determined by the cross-validation method. We empirically search *p* in {10, 20,…, 100} and *K* in {30, 40,…, 120}. Figure 5 plots the mean accuracy of DASRC with varying *p* and *K*, while using DE features. From Figure 5, we can see that DASRC can achieve stable performance with small *p* and *K*. The best accuracies are achieved, in general, when *p* is great than 60 and *K* is great than 80. This result indicates that the proposed DASRC can exploit the common knowledge in a relatively low dimensional subspace. Based on the results in Figure 5, the subspace dimension and dictionary size are suggested to be set to 60 and 80, respectively.

**Figure 6:**
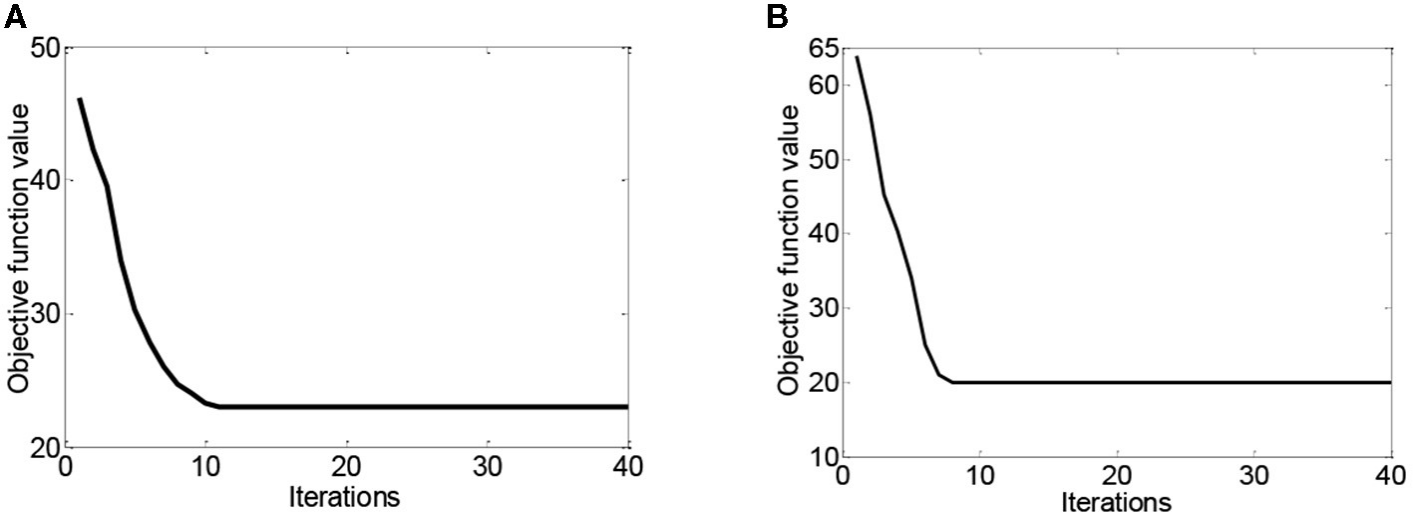
The convergence curves of DASRC on two scenarios, **(A)** cross-subject on SEED, **(B)** cross-dataset on DEAP→ SEED.

The domain adaptation methods often produce extra computational overhead. Figure 6 plots the convergence curves of DASRC in cross-subject and cross-dataset scenarios while using DE features. From this figure, we can see that DASRC can converge within a small number of iterations. Thus, we can set the iteration bound to 40.

## Conclusion

In this study, the DASRC model is proposed, which solves the EEG-based emotion classification across different subjects and datasets. Three criteria are considered to jointly learn sunspace and shared dictionary in DASRC. The local information preserved criterion is exploited to project samples in SD and TD into the shared subspace, where both PCA and Fisher criteria are exploited to transform discriminative knowledge through the shared dictionary. Experimental testing using SEED and DEAP datasets demonstrates the effectiveness of DASRC for dealing with the domain discrepancy for EEG-based emotion classification. For future work, we will explore more local preserved strategies in domain adaptation dictionary learning, such as local salience information. In addition, we will study the semi-supervised domain adaptation scenario, in which the unlabeled samples in TD rather than limited labeled samples participate in the model training. How to prevent negative transfer will also be considered in the next stage of work.

## Data Availability Statement

Publicly available datasets SEED and DEAP were analyzed in this paper. These data can be found in the following links, respectively: https://bcmi.sjtu.edu.cn/home/seed/ and http://www.eecs.qmul.ac.uk/mmv/datasets/deap/.

## Author Contributions

TN, JX, and SW conceived and designed the proposed model. YN and JX performed the experiment. TN and SW wrote the manuscript. All authors read and approved the manuscript.

## Conflict of Interest

The authors declare that the research was conducted in the absence of any commercial or financial relationships that could be construed as a potential conflict of interest.

## Publisher's Note

All claims expressed in this article are solely those of the authors and do not necessarily represent those of their affiliated organizations, or those of the publisher, the editors and the reviewers. Any product that may be evaluated in this article, or claim that may be made by its manufacturer, is not guaranteed or endorsed by the publisher.
